# Gender effects in spatial orientation: cognitive profiles and mental strategies

**DOI:** 10.1002/acp.1000

**Published:** 2004-07

**Authors:** Andrea Bosco, Anna M Longoni, Tomaso Vecchi

**Affiliations:** 1 Dipartimento di Psicologia, Università di BariItaly; 2 Dipartimento di Psicologia, Università di Roma ‘La Sapienza’Italy; 3 Dipartimento di Psicologia, Università di PaviaItaly

## Abstract

Experimental evidence and meta-analyses offer some support for gender-related differences in visuo-spatial ability. However, few studies addressed this issue in an ecological context and/or in everyday tasks implying spatial abilities, such as geographical orientation. Moreover, the relation of specific strategies and gender is still unclear. In the present investigation, we compared men and women in a newly designed battery of spatial orientation tasks in which *landmark*, *route* and *survey* knowledge were considered. In addition, four visuo-spatial working memory (VSWM) tasks were presented. Significant differences favouring men in VSWM tasks were reported, supporting existing evidence. However, men and women did not significantly differ in orientation tasks performance. The patterns of correlation between working memory and spatial orientation tasks indicated that men and women used somewhat different strategies in carrying out the orientation tasks. In particular, active processes seem to play a greater role in females' performance, thus confirming the importance of this variable in interpreting gender effect in VSWM tasks. Altogether, results indicate that gender effects could well result from differences in cognitive strategies and support data indicating that adequate training could reduce or eliminate them. Copyright © 2004 John Wiley & Sons, Ltd.

Gender effects on cognitive abilities have been largely investigated in the past. In particular, differences in visuo-spatial abilities have been reported and confirmed by experimental evidence (see Halpern, 2000; Maccoby & Jacklin, 1974; Richardson, 1991) and meta-analytic studies (Linn & Petersen, 1985; Voyer, Voyer, & Bryden, 1995). Several hypotheses have been put forward to explain these findings either focusing on biological factors such as hormones (e.g. Broverman et al., 1981; Kimura, 1999) or genetic influences (e.g. Dawson, 1972; Kimura, 1999). More recently, several authors highlighted the importance of socio-cultural factors on women’ performance in visuo-spatial tasks (e.g. Baenninger & Newcombe, 1989; Caplan, Crawford, Hyde, & Richardson, 1997; Richardson, 1994), showing significant effects of training and cognitive strategies.

Further research showed that gender effects in visuo-spatial tasks are not homogeneous: the characteristics of the task may induce the magnitude—sometimes the existence—of differences between men and women. Within a working memory framework, Vecchi and Girelli (1998) explored gender differences in visuo-spatial tasks that either required memorizing (passive task) or manipulating and transforming (active task) visuo-spatial information. Their results showed that gender effects, favouring men, were significantly more pronounced in active tasks. In general terms, the different theoretical approaches converge on the importance of dissociating memory and processing functions. The existence of different subsystems is consistent with traditional accounts of working memory (e.g. Baddeley, 1986; Logie, 1995) and it is crucial in explaining the involvement of central/coordinating functions in different tasks (Cornoldi & Vecchi, 2003). The distinction between passive and active processes in working memory proved to be useful also in interpreting individual differences (see Cornoldi & Vecchi, 2003).

For what concerns ecological studies results related to gender differences are not so straightforward. Methodological difficulties have often precluded the development of adequate experimental procedures: data are not always consistent. Ecological studies have been developed mostly in relation to spatial orientation ability, offering the possibility to investigate visuo-spatial processes in an everyday context and to design tasks associated to everyday activities. In spatial/geographical orientation an involvement of VSWM processes has been hypothesized and recently we proved a relationship between more general, undifferentiated visuo-spatial abilities and specific, frequentlyused orientation abilities (Bosco, Longoni, Rossi Arnaud, & Vecchi, 2001). Thus, investigation on spatial orientation seems to offer a ground, which could foster the assessment of gender differences in visuo-spatial functions within an ecological framework.

Although several studies have been carried out, a clear pattern of results did not emerge. Lawton and colleagues (Lawton, 1994; Lawton, Charleston, & Zieles, 1997) reported significant gender differences, favouring men, in different *wayfinding* tasks. By contrast, Olaughlin and Brubaker (1998) did not report differences in a mapping task. However Dabbs, Chang, Strong, and Milun (1998) showed that differences in the strategies used to carry out orientation tasks often emerged and could explain non significant data (e.g. Malinowski, 2001). Recently, the effect of mental speed was also proposed to explain gender effects as an important factor modulating cognitive strategies (e.g. Loring-Meier & Halpern, 1999; Scali, Brownlow, & Hicks, 2000). Altogether, these data do not provide consistent evidence for gender effects in visuo-spatial everyday tasks. However they do point to the importance of understanding more precisely the mechanisms underlying performance in orientation tasks, and specifically the role of cognitive strategies and individual differences. In addition, available data indicated that gender effects are not modulated by performance factors such as scoring procedures or time limitations in laboratory tasks (such as mental rotation of abstract figures, e.g. Masters, 1998). However gender differences could be eliminated by an appropriate instructional training (Kass, Ahlers, & Dugger, 1998) in an ecological task requiring comparison of orientation angles. Similarly, experience could play a major role in maximizing—or minimizing—gender effects (Voyer, Nolan, & Voyer, 2000). Recently, Vecchi (2001) has discussed the role of strategic factors in explaining gender differences. He suggested the existence of both biological and sociocultural/strategic factors in explaining individual differences in visuo-spatial abilities with possibly a greater role of ‘nurture’ (as opposed to ‘nature') in determining gender differences. This argumentation confirmed the importance of using spatial orientation tasks as a measure of more general visuo-spatial abilities and strengthened the need for ecological measures to be used in controlled settings.

Cognitive profiles and strategies could play a major role in interpreting available data and additional evidence is required to evaluate their impact on cognitive performance of men and women. The present study addressed these issues. Following a distinction originally proposed by Siegel and White in 1975, a set of spatial tests tapping *landmark, route* and *survey* knowledge was built up. These tasks have been proposed in association with four specific VSWM tasks designed to imply different processes in the system, sequential vs. simultaneous as well as passive vs. active. (Pazzaglia & Cornoldi, 1999; Vecchi & Cornoldi, 1999).

## EXPERIMENT

The aims of this investigation were i) to develop an original experimental procedure to evaluate different aspects of spatial orientation while maintaining a high ecological validity; ii) to compare patterns of correlation between orientation and working memory tasks in men and women; iii) to evaluate gender differences in the cognitive profiles of good and poor orienters in order to improve our understanding of the different strategies used; and finally iv) to interpret our findings within a general structure of VSWM.

## METHOD

### Subjects

One hundred and seven young adults (54 women) participated in this study. Age ranged between 18 and 36 years (mean age = 22.5). Participants were psychology students of the University of Rome and for their participation received a money voucher that could be used in a local bookseller.

### Materials and procedure

Four VSWM and eight orientation tasks were used.

#### VSWM tasks

1. Jigsaw puzzle span task (JP) (Richardson & Vecchi, 2002). Subjects were presented with numbered fragments of a picture of a common use object; all the fragments were presented to the subjects at the same time. They had to solve the puzzle by writing down the corresponding numbers on a response grid, without moving the pieces. The puzzles were presented at increasing levels of complexity consisting of 4, 6, 9, 12, and 15 fragments. The span value represents the level of complexity reached by the subjects.
2. Mental pathway task (MP) (Vecchi & Cornoldi, 1999). Participants had to follow pathways made of statements of direction in matrices of different complexity. A combination of matrix size and number of statements defined the overall span levels of complexity. For example level 1 (practice) comprised a 2 × 2 matrix with one statement, level 2 comprised a 2 × 2 with two statements, level 3 a 3 × 3 with three statements, level 4 a 3 × 3 with five statements, level 5 a 4 × 4 with four statements and so forth. The statements were left, right, forward, backward, the starting position was always the left inferior corner of the matrix. Participants were given sheets of paper on which matrices were represented, asked to follow the instructions and to mark on the matrix the final position of the pathway. The span level represents the level of complexity reached by the subjects.
3. Visual Pattern test (VP) (Della Sala, Gray, Baddeley, & Wilson, 1997). Matrices of various shapes were designed with an increasing numbers of white and black squares. Participants were presented with a matrix for two seconds, and asked to memorize the configuration. Immediately after they were asked to reproduce the pattern of black squares on a completely blank matrix of the same shape. The span level represents the highest number of black squares correctly recalled.
4. Corsi span test (CS) (Milner, 1971). The material consisted of a wooden board comprising nine blocks arranged in random positions. In each trial, subjects had to reproduce the sequence of positions previously shown by the experimenter. The length of the sequence increased on each trial. Two was the minimum length of the sequence. Three sequences were presented for each length. The span level represents the length of the longest sequence correctly reproduced.

#### Spatial orientation tasks

**Figure 1:**
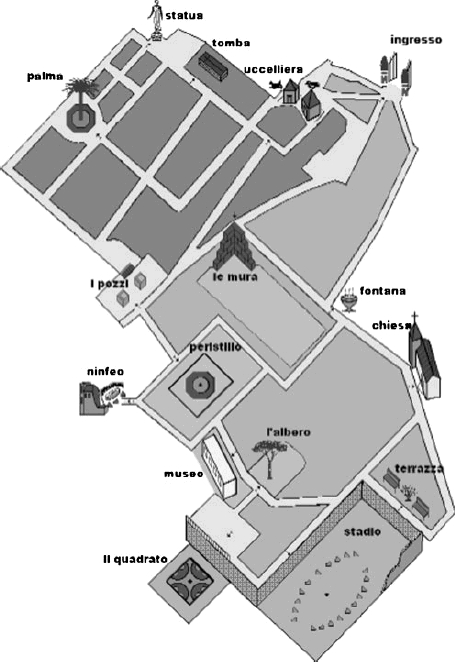
The schematized map of Palatino that has been used for the orientation tasks

In order to investigate spatial orientation abilities in an ecological context we decided to design a battery of tests based on a map-learning procedure. We prepared a simplified map of the Roman Palatino; an archaeological site open to visitors located on the hill where the legend tells Romolo founded Rome in 754 BC. Figure 1 shows the map including 16 landmarks. The structure of the experimental map and distances between landmarks are comparable to that of the real area. Each landmark is coupled with a little arrow indicating the imaginary position an observer should occupy when looking at the related landmark.

#### Landmark knowledge tasks

Landmark knowledge involves memory for the visual characteristics of the aspects of the environment chosen as landmarks. Each—landmark—could be remembered/recognized either as a single informational unit or within its environmental context.

1. Landmark recognition (L1): Sixteen triplets of stimuli were prepared. Within each triplet, each landmark is presented together with two incorrect alternatives and participants had to identify the correct picture. Performance is evaluated in terms of number of correct responses.
2. Landmark and surrounding recognition (L2): Sixteen triplets of stimuli were prepared. Within each triplet, each landmark and the surrounding area of the map are presented together with two alternatives in which only the surroundings were incorrect. Participants had to identify the correct picture. Performance is evaluated in terms of number of correct responses.

In light of theoretical assumption (no empirical evidences were still collected on it), these tasks should involve essentially visual—passive and simultaneous—processing.

#### Survey knowledge tasks

Survey knowledge involves a sort of map-like representation of the environment that integrates routes into a network of relationships between locations. It allows the navigator to localize places that are not perceptually available and to plan alternative routes for going from one location to another one.

1. Map completion (S1): An empty map is presented together with the complete list of the landmarks. Participants had to posit the 16 landmarks in the map. Performance is evaluated considering if the replacement was within a circle around the correct position. In particular, we assigned two points for each correct replacement within a circle of 2 cm diameter and one point for correct replacement within a 3 cm diameter circumference.
2. Map section rotation (S2): Eight experimental stimuli showing the spatial relations among three landmarks were designed. Within each trial, four alternatives were presented each including the same three landmarks in different spatial relations. Performance was evaluated in terms of number of correct trials.
3. Euclidean distance judgement (S3): Eight items were prepared requiring to estimate Euclidean overall distance between a designated landmark and three alternatives. Participants had to identify the longest distances. Performance was evaluated in terms of number of correct trials.

In terms of active/passive and simultaneous/sequential processing the Map Completion task should highly require passive and simultaneous processing during the recovery of spatial information. The Map Section Rotation task should involve passive and simultaneous processing together with active processing involved in mental rotation. Finally, the Euclidean distance judgement task should entail passive and sequential processing together with active processing due to the need to compare consecutively couples of distances.

#### Route knowledge tasks

Route knowledge involves the learning of a sequence of instructions about how to get from one location to the next and allows representing spatial information in an egocentric perspective. It requires the navigator to reorient to a new set of reference points when moving between different areas.

1. Route recognition (R1): Eight trials were prepared each including a triplet of described pathways between two designated landmarks. Participants had to identify the correct one and performance was evaluated in terms of number of correct trials.
2. Wayfinding (R2): Eight trials were prepared each requiring the participants to follow a described pathway and finally indicate the arrival point choosing the correct one among three alternatives. Performance was evaluated in terms of the number of correct trials.
3. Route distance judgement (R3): This task requires evaluating the route distance between a designated landmark and three other positions. Eight trials were prepared and performance was evaluated in terms of number of correct trials.

All together Route tasks should require essentially passive and sequential processing during the recovery of spatial information. In addition, they should entail very active processing due to the need to update spatial information during the imagined pathway.

Participants were tested in two phases. In the first one, they studied the map for 10 min. Following the study session, each participant was tested on the eight orientation tasks. This phase lasted about 1 h. In the second phase (2–3 days later), participants were tested on the four VSWM tasks. The latter phase lasted about 45 min. Participants were tested individually in both phases. The task presentation order was counterbalanced across subjects within each phase.

## RESULTS AND DISCUSSION

The first step in analysing the data was to determine if there were gender differences in the 12 tasks considered. Two MANOVAs were performed with gender as factor and VSWM and orientation tasks as dependent variables, respectively. Values were standardized (with mean equal to 50 and standard deviation equal to 10) to adjust for unequal measure units.

**Figure 2:**
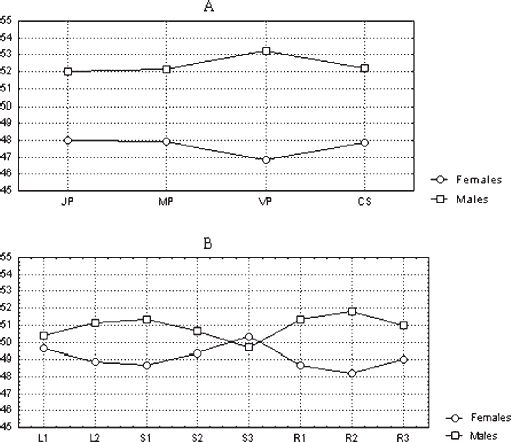
Profiles of women and men's performance in (A) the four VSWM tasks and (B) the eight orientation tasks. Values are reported in T points (mean = 50 and standard deviation = 10)

VSWM varied significantly with gender in terms of multivariate test, *Rao's R* (4, 102) = 3.41, *p<0.05,* mean (standard deviation) of women's Mahalanobis D^2^ = 3.61 (2.18) and men's Mahalanobis *D^2^ =* 4.32 (2.93). Univariate tests for each VSWM measure were also significant: JP F(1, 105) = 4.40, *p <* 0.05; MP F(1, 105) = 5.02, *p <* 0.05; VP F(1, 105) = 11.97, *p <* 0.001; CS F(1, 105) = 5.26, *p <* 0.05 (see Figure 2A). In summary, the multivariate gender effect was about 30% of Mahalanobis *D^2^* standard deviation, the univariate gender effects ranged from 30 and 50% of T scores standard deviation (in both cases favouring men).

The MANOVA carried out on the eight orientation tasks showed that despite the difference of means favouring men in almost every task (see Figure 2B) neither the multivariate test nor the univariate tests showed significant differences between men and women.

The second step in our analyses was to compare, in both genders, the involvement of VSWM in orientation task's performance. Two series of step wise forward multiple regression analyses—one for men and the other for women—were performed. Each analysis considered as *criterion* one out of eight orientation tasks and, in addition, an overall orientation ability measure consisting in a standardized sum of raw scores for each orientation task. Predictors in each analysis were the VSWM tasks. The results are reported in Table 1.

**Table 1:** Results of two series of multivariate regression analyses carried out on the eight orientation tasks with the four VSWM tasks as predictors, divided by gender

Orientation tasks	Females (*N* = 54)							Males (*N* = 53)						
	Beta values				*R*^2^	*F*(*df*)	*p*	Beta values				*R*^2^	*F*(*df*)	*p*
	JP	MP	VP	CS				JP	MP	VP	CS			
L1	0.34*	–	–	–	0.11	6.76(1, 52)	0.012	0.32*	–	–	–	0.10	5.72(1, 51)	0.020
L2	0.18	0.18	–	–	0.09	2.50(2, 51)	0.091	0.34*	–	–	0.17	0.20	6.12(2, 50)	0.004
S1	0.27*	0.22	–	–	0.16	5.02(2, 51)	0.010	–	–	0.40**	0.22	0.27	9.15(2, 50)	0.000
S2	–	0.19	–	–	0.04	2.07(1, 52)	0.156	0.33*	–	–	–	0.11	6.32(1, 51)	0.015
S3	–	–	–	–	–	–	–	0.20	–	–	–	0.04	2.10(1, 51)	0.153
R1	–	–	–	0.21	0.04	2.30(1, 52)	0.135	–	–	0.27*	0.29*	0.21	6.72(2, 50)	0.002
R2	0.17	–	–	–	0.03	1.48(1, 52)	0.229	–	0.25	0.21	–	0.14	3.95(2, 50)	0.025
R3	−0.15	0.19	–	–	0.04	1.09(2, 51)	0.341	0.20	–	–	–	0.04	2.14(1, 51)	0.149
Overall	0.28*	0.29*	–	–	0.21	6.75(2, 51)	0.002	0.20	0.14	0.29*	0.18	0.38	7.27(4, 48)	0.000

* Significant at *p*<0.05;

** *p*<0.01.

*R^2^* values of men regressions were higher than in women analyses *(R^2^* related to the overall measure of orientation was 0.38 and 0.21 respectively). This trend was confirmed in every orientation task. VSWM ability explains a larger part of variance in men than in women. Moreover, the structure of relationship between VSWM in S1 (Map completion task) men and women showed a different pattern of significant predictors: the largest *beta* values corresponded to the *active* tasks in women (i.e. JP and MP). By contrast, men showed the largest coefficients in the passive tasks (i.e. VP and CS). Finally, no route tasks obtained significant *R^2^* in women, in contrast *R^2^* of R1 and R2 tasks were significant in the sample of men. These findings suggested that: i) VSWM was more strongly related to orientation ability in men than in women; ii) when VSWM was involved in orientation ability both in men and women, the structure of such relationship was gender-related.

**Figure 3:**
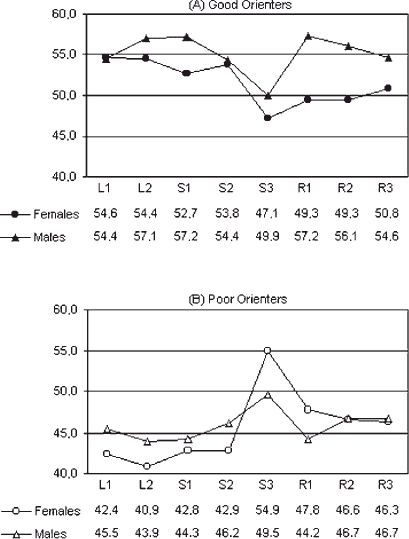
Profiles of (A) good and (B) poor orienters as a function of gender in eight orientation tasks. Values are reported in T points (mean = 50 and standard deviation = 10)

In order to evaluate any difference in the cognitive profiles of men and women's orientation ability, the eight spatial orientation tasks were considered in two k-means cluster analyses separately for men and women. This statistical technique started with *k* random (or selecting the most different *k* cases) clusters, and then moved objects between those clusters with the goal to (1) minimize variability within clusters and (2) maximize variability between clusters. In the present study the *selected k cases* procedure was preferred. An exploratory approach of k-means cluster analysis was chosen to evaluate a number of different models. This approach consists in repeating cluster analysis *n* times adding a unity to the previous *k* value considered. Starting point was *k = 2,* the analysis was completed with *k =* 6 (this value was suggested by the findings of a previous study, A. Bosco, unpublished dissertation, 1999). Different *criteria* were considered in choosing the best model. The magnitude of the *F* values from the analysis of variance performed on each dimension is an important indication of how well the respective variable discriminates between clusters. Another important indication comes from the theoretical evaluation of the model with respect to previous findings. Finally, the parsimony of number of groups in choosing the best model should be considered. The *k = 2* model was (both in women and men analyses) the best cluster solution^1^. Observing the means of each group it emerges that subjects were clustered in terms of mean level of performance: we labelled these subjects as *poor* (N_women_ = 22, N_men_ = 24) and *good* (N_women_ = 32, N_men_ = 29) *orienters.* Figure 3 shows that the different ability groups seem to behave differently as a function of gender.

In line with this consideration two series of one-way between-subjects ANOVAs were performed on the eight orientation tasks separately for the two ability groups. Independent variable was *gender.* Analysing separately good and poor orienters, differences between men and women seem to emerge. In the good orienters analysis men showed a higher performance than women in three different tasks: one referred to the ability in map completion and two referred to the ability in imaging a route (S1, R1 and R2 tasks, see Table 2A). By contrast, in the poor orienters analysis, females showed a comparable level of performance with respect to men in almost every task, while they performed better than men in Euclidean distance judgement, a metrical task (S3 task, see Table 2B).

**Table 2:** Results of ANOVAs as a function of gender separately for (A) good and (B) poor orienters on the eight orientation tasks

Dependent variables	Effect	Error	*F*	*p*-level	Means	
					Females	Males
(A) Good orienters						
L1	0.41	74.31	0.01	0.941	54.61	54.44
L2	106.72	29.41	3.63	0.062	54.41	57.06
S1	314.71	41.94	7.50	0.008	52.66	57.21
S2	4.79	29.46	0.16	0.688	53.80	54.36
S3	117.56	102.21	1.15	0.288	47.09	49.87
R1	959.74	87.05	11.03	0.002	49.29	57.24
R2	696.37	88.29	7.89	0.007	49.31	56.08
R3	213.09	94.05	2.27	0.138	50.84	54.58
(B) Poor orienters						
L1	108.23	72.65	1.49	0.229	42.39	45.46
L2	105.12	93.14	1.13	0.294	40.91	43.93
S1	23.93	101.91	0.23	0.630	42.85	44.29
S2	129.03	144.99	0.89	0.351	42.86	46.21
S3	331.83	85.52	3.88	0.055	54.91	49.53
R1	142.72	68.77	2.08	0.157	47.77	44.24
R2	0.10	86.10	0.00	0.972	46.60	46.69
R3	1.47	87.78	0.02	0.898	46.34	46.70

Results showed that the unclear gender effect in spatial ecological tasks was probably due to the effect of *merging* in the same sample of people that showed differences not only in terms of *level of performance,* but also in terms of *strategies* or *styles* of task solution. Gender differences may be masked by individual differences.

## CONCLUSION

The first aim of this study was to provide a battery of tasks proving to be useful in investigating orientation abilities in an ecological context. The characteristics of these tasks are as follows: 1) they had to be related to the relevant theoretical concepts in this field, namely landmark, route and survey knowledge, as originally proposed by Siegel and White (1975). 2) The Palatino map proved to be the ideal ground for developing a battery of sensible tools for investigating individual differences. It is rather different from other artificial maps that have been used in the literature, such as the one designed by Thorndyke and Stasz (1980) with respect of two characteristics: the distinctive visual characteristics of the different landmarks and the irregular not grid-like nature of the map. 3) All tasks require recognition: this procedure increased the ecological value of these tasks, maintaining at the same time the possibility of differentiating among groups. In addition, the characteristics of the material make it possible to hypothesize a future use with people who already experience a reduction in their cognitive abilities, such as patients affected by Alzheimer-type dementia, or people deserving special attention like elderly people. It has been repeatedly suggested that visuo-spatial deficits as well as orientation and geographical abilities could well predict early signs of neurological deterioration (e.g. Beatty & Bernstein, 1989; Kaskie & Storandt, 1995). However, a battery of orientation tasks that could address both clinical and theoretical needs is not, at present, available.

In order to evaluate the contribution of VSWM on orientation performance—namely the ability to use map information in a wide sample of orientation tasks such as map completion, wayfinding, map rotation, Euclidean and route distance judgement—a set of VSWM tasks involving both active and passive processing was used. Moreover we analysed women and men sub-samples in order to evaluate differences, if any, in the structure of such relationship. By considering the overall performance in the eight orientation tasks, results indicated a relationship between these two sets of abilities. However, the percentage of explained variance predicted by VSWM abilities is significantly higher for men than for women. This result is of particular importance since empirical evidence was not available on the contribution of VSWM in orientation tasks. Although the involvement of VSWM abilities in orientation tasks is less relevant in women than in men, active processes seem to play an important role. This result strengthened the relationship between visuo-spatial active processes and female gender, as previous studies supported (e.g. Paivio & Clark, 1991; Vecchi & Girelli, 1998). From a theoretical point of view, data confirmed the importance of considering the characteristics of the task as an essential variable in interpreting working memory functions. Each task could well be defined in terms of amount of active manipulation required and this variable influences the magnitude of individual differences (see Cornoldi & Vecchi, 2003).

Moreover, present evidence suggests that lower abilities could not reflect limitations in the active processing component but rather the choice of visuo-spatial strategy involving an overload of active resources or simply an incorrect selection of the best strategy for each type of task. Our data also suggest that gender differences do emerge in association with laboratory tests but are less evident in ecological tasks. This could partially explain the adoption of different strategies in the female populations: such strategies are overall less efficient but do not determine critical limitations in everyday life.

Our results also pointed out the importance of considering gender effect while investigating visuo-spatial abilities since different patterns of performance between men and women could result in minimizing overall cognitive differences thus determining non significant results. This shows the need to analyse gender effects in conjunction with cognitive strategies as for the performance of poor and good orienters. In several tasks, poor and good orienters did show a partially different pattern in men and women. This pattern is clearly compatible with the adoption of different strategies in relation with gender determining significant differences in performance. Results showed an interesting pattern of relationship between poor and good orienters and gender: three tasks (two route tasks and the map completion task) showed gender-related differences, favouring men, in good orienters, whereas the Euclidean distance judgement task showed an opposite pattern with poor orienters. The nature and characteristics of men and women’ strategies cannot be inferred from the present study and should be further investigated in future research. However present data confirm the importance of interpreting gender differences not only in terms of specific cognitive capacities but also with relation to more general metacog-nitive issues.

Our results highlighted a set of differences between men and women: 1) VSWM predict orientation ability, better in men than in women. 2) The orientation performance of women is more accurately predicted by the tasks involving active processing, indicating that it is critical in women's visuo-spatial performance. 3) Gender-related differences emerge between men and women when different analyses for low and high ability groups are performed. In particular male good orienters show a higher performance than women on both route and survey tasks. 4) Gender-related differences in VSWM are confirmed: our results confirm the previous findings.

In conclusion, visuo-spatial abilities could play an important role in the execution of orientation tasks. However, only a limited percentage of variance in orientation tasks can be explained by VSWM tasks and it is necessary to hypothesize a greater involvement of other components of working memory. Moreover, the involvement of VSWM in orientation abilities seems to be related to gender differences. Men tend to use visuo-spatial abilities when orienting in the environment to a greater extent than women do; this finding is confirmed by results indicating a difference between men and women in the good orienters ability group. These results highlighted that cognitive strategies may both modulate cognitive abilities and help interpret gender differences. The critical role of mental strategies in determining gender differences in visuo-spatial tasks has been recently pointed out by Vecchi (2001) when analysing the nature of male's advantage in an active visuo-spatial task. The interpretation of individual differences in terms of selection and adoption of different cognitive strategies does highlight the role of metacognitive abilities in determining the level of VSWM capacity (see Cornoldi & Vecchi, 2003) and, from a theoretical perspective, the importance of considering the working memory system as a whole. A visuo-spatial task can be carried out through the active involvement of more than a single component of the system and thus it is particularly important to understand not only dissociation between separable components but also interaction and coordination between such components.
